# Ribosome dysfunction in osteoarthritis

**DOI:** 10.1097/BOR.0000000000000858

**Published:** 2021-11-25

**Authors:** Guus G.H. van den Akker, Marjolein M.J. Caron, Mandy J. Peffers, Tim J.M. Welting

**Affiliations:** a Laboratory for Experimental Orthopedics, Department of Orthopedic Surgery, Maastricht University; b Laboratory for Experimental Orthopedics, Department of Orthopedic Surgery, Maastricht University Medical Center, Maastricht, the Netherlands; c Institute of Life Course and Medical Sciences, University of Liverpool, Liverpool, UK

**Keywords:** osteoarthritis, protein translation, ribosome, rRNA, snoRNA

## Abstract

Translation of genetic information encoded within mRNA molecules by ribosomes into proteins is a key part of the central dogma of molecular biology. Despite the central position of the ribosome in the translation of proteins, and considering the major proteomic changes that occur in the joint during osteoarthritis development and progression, the ribosome has received very limited attention as driver of osteoarthritis pathogenesis.

**Recent findings:**

We provide an overview of the limited literature regarding this developing topic for the osteoarthritis field. Recent key findings that connect ribosome biogenesis and activity with osteoarthritis include: ribosomal RNA transcription, processing and maturation, ribosomal protein expression, protein translation capacity and preferential translation.

**Summary:**

The ribosome as the central cellular protein synthesis hub is largely neglected in osteoarthritis research. Findings included in this review reveal that in osteoarthritis, ribosome aberrations have been found from early-stage ribosome biogenesis, through ribosome build-up and maturation, up to preferential translation. Classically, osteoarthritis has been explained as an imbalance between joint tissue anabolism and catabolism. We postulate that osteoarthritis can be interpreted as an acquired ribosomopathy. This hypothesis fine-tunes the dogmatic anabolism/katabolism point-of-view, and may provide novel molecular opportunities for the development of osteoarthritis disease-modifying treatments.

## INTRODUCTION

The dogmatic long-standing view on the molecular pathobiology of osteoarthritis is that of an imbalance between anabolism and catabolism of tissues and cells of the joint [1]. From a plethora of studies reported over the past decades, it has become overwhelmingly clear that the low turn-over homeostatic balance of a healthy joint becomes compromised in osteoarthritis and a net loss of tissue occurs. This is caused by a compromised balance between anabolic reparative capacity and catabolic degenerative activity, resulting in the destruction of anatomical joint function. A major part of the extracellular matrix of joint tissues and many of the molecules involved in anabolic and catabolic cellular events are proteins. Osteoarthritis-related aberrations in gene and protein expression are widely reported in the literature. However, the central cellular hub that critically catalyses the biosynthesis of these proteins from dedicated gene transcripts; the ribosome, has been largely neglected in osteoarthritis research. In addition to a variety of genetic and epigenetic regulatory mechanisms of protein expression, it has now become clear that regulation of protein expression also takes place at the level of the ribosome itself [2]. Environmental, developmental and pathological conditions are all able to influence the protein translation characteristics of the cellular ribosome pool [3], including ribosome heterogeneity as a mechanism for preferential translation [4]. This has major consequences for total protein translation capacity but also for the synthesis of specific proteins involved in development, homeostasis and pathology. 

**Box 1:**
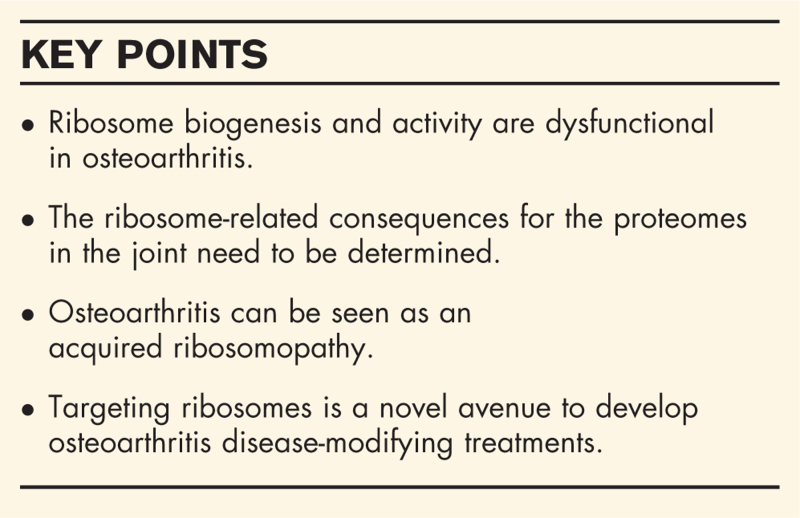
no caption available

## THE RIBOSOME IN OSTEOARTHRITIS

### Disrupted ribosome biogenesis in osteoarthritis

**FIGURE 1:**
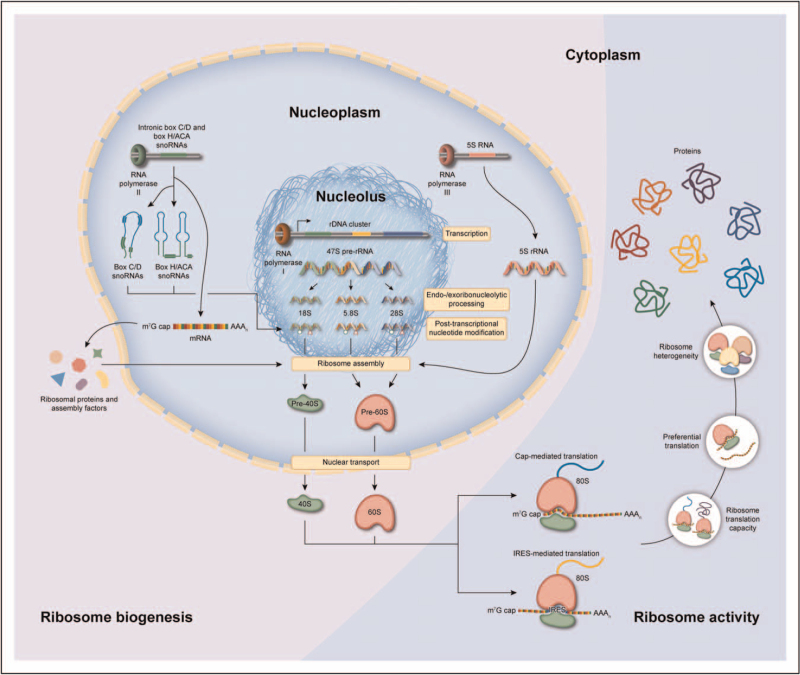
An overview of ribosome biogenesis and activity. 47S prerRNA is transcribed in the nucleolus from rDNA clusters by RNA polymerase I. The 47S precursor is simultaneously processed by endoribonucleases and exoribonucleases and posttranscriptionally modified by snoRNPs. These snoRNPs consist of an enzyme, accessory proteins and box C/D or box H/ACA snoRNAs that guide site-specific 2’-*O* ribose methylation or pseudouridylation of rRNA nucleotides. These canonical snoRNAs originate from intronic regions of RNA polymerase II-transcribed mRNAs and are liberated by the splicing machinery and subsequent processing. Together with mature rRNA, ribosomal proteins assemble into the small 40S and large 60S ribosomal subunits. This highly coordinated process requires additional assembly factors. After nuclear export, the 40S subunit can form a 43S preinitiation complex together with eukaryotic translation initiation factors that recognize the m^7^G cap of mRNAs and initiate protein translation after recruitment of the 60S large subunit, resulting in the formation of an active 80S ribosome. In addition to cap-mediated translation, Internal Ribosome Entry Sites (IRESs) can mediate direct recruitment of the ribosome to a translation start site. This process is of paramount importance under cellular stress conditions, where cap-mediated translation is generally inhibited. Ribosome activity is tightly regulated by a multitude of signalling pathways (e.g. AKT/mTOR, TGF/BMP, FGF), and other important factors are energy status and amino acid availability for aminoacyl-tRNA formation. The most rate-limiting step of ribosome activity is translation initiation by eIF4E, which is counteracted by 4E-BP1. In addition, ribosomes can preferentially translate a certain mRNA because of specific (IRES) trans-acting factors. To add to the complexity, the ribosome can generate multiple protein variants from a single mRNA, when more than one or alternative translation initiation sites are present (e.g. in VEGF, MYC and FGF2 mRNAs). Ribosome core protein composition and rRNA posttranscriptional modification levels can vary [4,10], which leads to heterogeneous ribosomes with distinct translational characteristics.

In order to build a ribosome, the cell is equipped with sophisticated mechanisms that support the biogenesis of a ribosome in a highly orchestrated and regulated manner (Fig. 1). Although the pathway of ribosome biogenesis is tightly integrated in the cell, the high complexity of this process makes it relatively vulnerable to aberrations that may result in pathological consequences. In the field of oncology, a wide variety of alterations in ribosome biogenesis are known to promote carcinogenesis [5–7], and a number of anticancer drugs target ribosome biogenesis pathways [8,9]. In osteoarthritis, however, the role of ribosome biogenesis is only beginning to be studied. Human ribosome biogenesis is initiated in the nucleolus and starts with the transcription of the 47S preribosomal RNA (rRNA) precursor by the dedicated RNA polymerase I transcription complex [10] (Fig. 1). There are ±200 copies of the *47S* gene spread over five chromosomes, forming the nucleolus organizer regions. Following transcription, the 47S prerRNA transcript is endoribonucleolytically and exoribonucleolytically processed into the 18S, 5.8S and 28S rRNAs, and a number of these processing steps take place co-transcriptionally. The 5S rRNA is transcribed separately in the nucleus by RNA polymerase III and imported in the nucleolus to be integrated into the ribosome biogenesis pathway. In ageing (a major risk factor for osteoarthritis), mouse bone marrow cells have been shown to have an increased rDNA copy number [11]. However, these rDNA copies have increased CpG methylation levels [11,12], probably leading to the observed reduction of rRNA expression [11]. Recent work demonstrated regulation of rRNA expression by a Chromobox 4 (CBX4)-dependent mechanism. This mechanism of nucleolar homeostasis protects against mesenchymal stem cell senescence and against murine osteoarthritis development [13]. In addition, cartilage ageing led to lower expression of the RNA component of mitochondrial RNA processing endoribonuclease (RMRP) in equine chondrocytes [14]. This small nucleolar RNA (snoRNA) is a key factor in the endoribonucleolytic processing of the 47S prerRNA [15] and provides an indication of age-related impairment of chondrocyte rRNA processing. This was confirmed in human chondrocytes, when RMRP snoRNA expression was found to be enriched in hypertrophic chondrocytes in a single-cell sequencing analysis of osteoarthritis cartilage [16^▪^,^17^]. Another key factor in the endoribonucleolytic processing of the 47S prerRNA is U3 snoRNA [18]. The expression of U3 snoRNA was reduced in human osteoarthritis cartilage and chondrocytes, as well as in murine joints in which experimental osteoarthritis [destabilization of the medial meniscus (DMM)] was induced [19^▪▪^]. Osteoarthritis-dependent inhibition of U3 snoRNA transcription was identified as one of the causes of reduced U3 snoRNA expression in osteoarthritis chondrocytes and resulted in a decrease of chondrocyte rRNA levels [19^▪▪^].

Except for 5S rRNA, all rRNAs are targets of snoRNA-mediated site-specific posttranscriptional modification (PTM) by 2’*O*-ribose methylation and pseudouridylation by fibrillarin and dyskerin, respectively. A total of 226 of these PTMs have been identified on human rRNAs [20]. A large family of canonical snoRNAs [active as small nucleolar ribonucleoprotein particles (snoRNPs)] ensures the site-directionality of these PTMs [21]. Differential expression of canonical snoRNAs was demonstrated in human ageing and osteoarthritis cartilage [22^▪▪^], in murine DMM joints [23], and in equine ageing cartilage [14]. Mechanistic analysis of the role of a number of these snoRNAs in chondrocyte biology demonstrated that SNORD26 and SNORD96A are involved in determining the chondrocyte phenotype [22^▪▪^] and SNORD32A, SNORD33 and SNORD35A in oxidative stress responses [24]. A great number of differentially expressed snoRNAs await further mechanistic studies and their consequences for rRNA PTM and ribosome function in cells from joints tissues need to be dissected. In this respect, our group mapped the rRNA PTM landscape in an in-vitro model for osteoarthritis chondrocytes and identified osteoarthritis-dependent changes in rRNA PTMs with consequences for the modus of ribosome translation initiation [25]. Differential expression of canonical snoRNAs was also detected in synovial fluid of early equine osteoarthritis [26], in the serum of DMM mice and horses [26] and in the serum of an anterior cruciate ligament injury cohort [27]. These studies may provide snoRNA-based biomarkers for musculoskeletal ageing and osteoarthritis development.

In addition to the rRNAs, the ribosome consists of 79 proteins [33 RPS (ribosomal protein small subunit) proteins in the 40S subunit and 46 RPL (ribosomal protein large subunit) proteins in the 60S subunit]. These proteins are imported into the nucleus and depending on the protein species and its position in the ribosome's biomolecular architecture, are assembled in a highly orchestrated sequence, which requires over 100 ancillary proteins to achieve this task [10,28]. This number excludes snoRNP-related proteins. The 40S and 60S ribosomal subunits are then transported to the cytoplasm and undergo several final maturation steps before they are ready to engage in the translation of mRNA into protein. Emerging evidence indicates that in osteoarthritis, the proteinaceous part of the ribosome's architecture may change. In recent single-cell sequencing work, it was demonstrated that increased RPS29 (ribosomal protein S29) expression in chondrocytes is associated with osteoarthritis progression [16^▪^]. The involvement of ribosomal protein expression in osteoarthritis was also highlighted in a recent single-cell sequencing study that investigated cross-talk between the synovium and cartilage in osteoarthritis [29]. A great number of ribosomal proteins were found to be differentially expressed in cell subpopulations of osteoarthritis synovium and osteoarthritis cartilage from damaged and nondamaged areas [29]. Although their association with functional ribosomes needs to be investigated, these observations at least highlight an interaction between the osteoarthritis disease stage, the chondrocyte phenotype, and the regulation of the expression of core ribosomal proteins. Indeed, recent work from our group demonstrated that in chondrogenesis, the expression of the chondrogenic transcription factor Sox9 regulates the expression of ribosomal proteins as well as proteins involved in the ribosome biogenesis pathway [30^▪▪^]. The osteoarthritis disease-dependent regulation of expression of core ribosomal subunits hints towards ribosome heterogeneity, which is a level of protein translational control that was recently discovered [31].

### Altered ribosome activity in osteoarthritis

The primary function of the ribosome is the translation of mRNAs into proteins (Fig. 1). Multiple studies have gathered compelling evidence of major proteomic changes in fluids, cells and tissues of the osteoarthritis joint [32–39]. For a long time, it was assumed that these changes originate from major mRNA transcriptomic changes only. An additional important level of epitranscriptomic regulation was later introduced by the identification of microRNA networks that control the translation of mRNAs involved in osteoarthritis development [40]. However, it was only recently demonstrated that protein synthesis in osteoarthritis is also regulated at the level of ribosome activity (Fig. 1). It was shown that ribosome protein translation activity was increased in human osteoarthritis chondrocytes, in a rat model for traumatic osteoarthritis, and in an IL-1β-dependent in-vitro chondrocyte model for osteoarthritis [41]. The identified mechanism behind this observation was a mammalian target of rapamycin complex 1 (mTORC1)-mediated inhibition of eIF4E-binding protein 1 (4E-BP1). As the activity of 4E-BP1 is rate-limiting for the activity of eIF4E, the cap-binding protein responsible for cap-dependent protein translation, this indicates that this mechanism primarily involves cap-mediated translation initiation. In concert with involvement of mTORC1 in cartilage homeostasis, mTOR activity was shown to be increased in osteoarthritis but was then linked to cartilage autophagy [42]. The finding that the mTORC1-mediated inhibition of 4E-BP1 precedes cartilage degeneration in rat osteoarthritis knees [43] strongly suggests its involvement in early osteoarthritis. In contrast to an overall increased level of chondrocyte protein translation activity, work from our group demonstrated that protein translation activity was reduced in chondrocytes isolated from end-stage knee osteoarthritis cartilage [19^▪▪^]. This was accompanied by lower levels of rRNA in osteoarthritis chondrocytes and chondrocytes treated with osteoarthritis synovial fluid. In addition, in a study comparing end-stage osteoarthritis cartilage with normal cartilage, it was found that expression of 4E-BP1 was higher in osteoarthritis cartilage, which is indicative of a reduction of translational activity [44^▪^]. Fibroblast growth factor (FGF) signalling [45] was described as another mechanism underlying a reduction in chondrocyte protein translation [46]. However, this mechanism was mTOR-independent. In addition, the recent single-cell sequencing work in chondrocytes demonstrated differential expression of translation initiation factors 4E-BP1 [in proliferative chondrocytes (ProCs)] and EIF4A1, EIF4A2, EIF4A3, EIF1 and EIF5 [in homeostatic chondrocytes (HomCs)] [16^▪^,^29^]. Interestingly, in an osteoarthritis serum biomarker study, patients with knee osteoarthritis had significantly lower serum levels of 4E-BP1, which was found be positively correlated with osteoarthritis pain intensity [34]. Together, this suggests that the mechanisms and involvement of deregulated ribosome protein translation activity in osteoarthritis chondrocytes are far more complex and depend on the stage of osteoarthritis progression and the chondrocyte phenotype, warranting further studies to dissect its complexity.

Rather than performing protein translation in a textbook manner, it has become clear that there is a large level of translational regulation that drives preferential translation of specific mRNAs [47]. Protein translation initiation can occur via multiple mechanisms and two well described mechanisms are cap-dependent translation and IRES-dependent translation (Fig. 1). Although cap-dependent translation is considered to constitute the majority of translation events, the cell preferentially uses IRES-dependent translation for the synthesis of many of its stress-related proteins. Indeed, ongoing work by our group demonstrates that in chondrocytes TNFα induces protein translation from the FGF1 IRES [48]. In addition, treatment of chondrocytes with TGFβ induced their protein translational activity [44^▪^,^49^] but skewed their preferential mode of translation toward cap-dependent translation [49]. The balance between cap-dependent and IRES-dependent translation is amongst others determined by specific snoRNA-mediated PTMs on the rRNA [18] and by expression of IRES-transacting factors (ITAFs). With the mapping of differential expression of snoRNAs [14,22^▪▪^,^23^,^26^,^50^] and rRNA PTMs [25], as well as high-resolution proteomics [30^▪▪^,^39^] in cells from joint tissues as a function of ageing [51,52] and osteoarthritis, it is expected that insight into this level of ribosome translation regulation will further unfold. The existence of other mechanisms of preferential translation in cell types from the joint is only starting to emerge. Recently ribosome profiling coupled to protein mass-spectrometry demonstrated that treatment of chondrocytic cells with IL-1β induced the preferential translation of proteins associated with inflammatory responses and oxidative stress [53^▪▪^]. IL-1β -induced preferential translation of osteoarthritis-related proteins in chondrocytes was suggested to be mediated by their 5′ untranslated regions [54].

### Future perspectives

It is becoming clear that alterations in ribosome biogenesis and ribosome function find their place in the molecular pathobiology of osteoarthritis. However, considering the incredible complexity of ribosome biogenesis and the many mechanisms by which ribosome activity and modus can be influenced, this field of osteoarthritis research is only just emerging. In discovery-driven approaches, we need to further chart the levels and identify the molecules by which ribosome biogenesis and function are being disturbed in osteoarthritis. Cellular stress signalling provoked by environmental factors like growth factors, cytokines, chemokines, damage-associated molecular patterns, senescence, metabolites, mechanosensing mechanisms and the extracellular matrix are all, in one way or the other, involved in osteoarthritis development and its progression. The osteoarthritis-related proteomic changes in the tissues of the joint induced by these pathological stress signalling events can all be candidates for regulation at the translational level by the ribosome. This can either be caused by changes in ribosome biogenesis, total protein translation capacity, or by mechanisms of preferential translation of mRNAs. An interesting connection between ribosome biogenesis and cellular stress is p53. P53 expression is upregulated in senescent and osteoarthritis chondrocytes [55,56]. In addition, p53 is a major regulator of ribosome biogenesis stress and p53 activation shuts down ribosome biogenesis at multiple levels [57]. This can lead to impairment of the total protein translation capacity with consequences for cartilage proteostasis [52]. Another ribosome-related stress factor relevant to chondrocytes is endoplasmic reticulum (ER) stress [58]. ER stress triggers the unfolded protein response (UPR). UPR activation has been demonstrated in osteoarthritis chondrocytes [59,60] and recently it was shown that ER stress and the UPR are specifically involved in the onset of experimental osteoarthritis but not in its progression [61]. Whether alterations in ribosome biogenesis are involved in osteoarthritis chondrocyte ER stress is currently unclear. However, ribosome translation activity can be connected to ER stress and the UPR via inactivation of eukaryotic translation initiation factor (eIF) 2α and inhibition of 80S ribosome assembly [58,62]. Recent evidence demonstrates that translation of catabolic proteins in chondrocytic cells is preferred in osteoarthritis-mimicking environments [53^▪▪^,^54^]. The finding that mechanisms of such preferential translation can also include heterogeneity of the cellular ribosome pool is an exciting development that may further unveil how cells in joints tissues can translationally respond to changes in their environment at the protein level (Fig. 1). However, the potential ribosome biogenesis-related and ribosome activity-related consequences for the proteomes of different tissue types from the joint still need to be determined.

## CONCLUSION

As outlined above, a slowly increasing amount of experimental evidence highlights that molecular mechanisms involved in ribosome biogenesis and ribosome activity are deregulated in osteoarthritis. The fact that we can find these aberrations from early-stage ribosome biogenesis, through ribosome build-up and maturation, up to preferential translation by the ribosome is fascinating. Underlying genetic factors do not seem to be the cause of these translational deficits. In contrast, ageing and many environmental factors are clearly connected to disturbances in ribosome biogenesis and ribosome activity in general, and also represent main risk factors for the development and progression of osteoarthritis. Classically, osteoarthritis has been explained as a disbalance between joint tissue anabolism and catabolism. Considering the current evidence collected in this article on osteoarthritis-related aberrations in ribosome biogenesis and ribosome function, we therefore hypothesize that osteoarthritis can be molecularly interpreted as an acquired ribosomopathy [63^▪^]. This hypothesis further fine-tunes the dogmatic anabolism/catabolism point-of-view by adding aberrations in total protein translation capacity and preferential translation to the molecular pathogenesis of osteoarthritis. This may provide novel molecular opportunities for the development of osteoarthritis disease-modifying treatments.

## Acknowledgements

*The authors thank Bobby Li (Sketchy Pipette) for graphical design support for the figure.*

### Financial support and sponsorship

*T.J.M.W. is funded through a grant from Stichting de Weijerhorst (Bewegen zonder Pijn) and grants from the Dutch Arthritis Foundation (LLP14 and 17-2-401). M.J.P. is funded through a Wellcome Trust Clinical Intermediate Fellowship (grant 107471/Z/15/Z).*

### Conflicts of interest

*There are no conflicts of interest.*

## REFERENCES AND RECOMMENDED READING

Papers of particular interest, published within the annual period of review, have been highlighted as:

▪ of special interest
▪▪ of outstanding interest
